# Combination immunotherapy with anti-CTLA-4 and interleukin-2 redirects regulatory T cells into tumor-draining lymph nodes and expands anti-tumor CD8+ T cells in the tumor microenvironment

**DOI:** 10.1186/2051-1426-2-S3-P97

**Published:** 2014-11-06

**Authors:** Joseph Broucek, Tasha Hughes, Erica Huelsmann, Eugene Lusciks, Graham Hill, Janet Zayas, Joseph Poshepny, Carl Ruby, Frederick Kohlhapp, Andrew Zloza, Howard Kaufman

**Affiliations:** 1 Rush University Medical Center, Chicago, IL, USA; 2 Rutgers Cancer Institute of New Jersey, New Brunswick, NJ, USA

## Background

Monotherapy with Ipilimumab (anti-CTLA-4 antibody) and monotherapy with IL-2 (T cell stimulating cytokine) are approved for the treatment of metastatic melanoma. Combination immunotherapy has been suggested as a more potent regimen but has not been sufficiently investigated. We hypothesized that this combination may enhance therapeutic responses through alteration of the effector CD8+ T cell to CD4+FoxP3+ regulatory T cell ratio.

## Methods

On day 0, C57BL/6 mice were challenged via intradermal injection with B16-F10 melanoma (120,000 cells). To track anti-tumor CD8+ T cell responses, pmel CD8+ T cells (specific against melanoma antigen gp100; 50,000 cells) were adoptively transferred via retroorbital injection. On days 3, 6, and 9 anti-CTLA-4 (100 µg in 100 µl or 100 µg IgG control) was administered via intraperitoneal injection. On days 4-8 IL-2 (100,000 units in 100 µl or 100µl PBS) was administered every 12 h. Tumor area was measured every 2-3 days until reaching 100 mm^2 ^or until mice were sacrificed for flow cytometry analysis of T cell responses in the tumor and tumor-draining lymph nodes.

## Results

Tumor growth was significantly reduced with combination anti-CTLA-4 and IL-2 treatment (average tumor area: 2mm^2 ^on day 14) compared to anti-CTLA-4 only, IL-2 only, and placebo treatment (14, 29, and 68 mm^2^, respectively, on day 14) (p < 0.01 for all comparisons). On day 4, regulatory T cells were decreased by 20% in the tumor with anti-CTLA-4 therapy alone or in combination with IL-2, compared to placebo. By day 10, regulatory T cells decreased by 80% in the tumor, but increased three-fold in the tumor-draining lymph nodes, compared to placebo (p < 0.01 for all comparisons). In the tumor microenvironment, anti-tumor (pmel) CD8+ T cells were increased 2-fold with IL-2 therapy alone or in combination with anti-CTLA-4, compared to placebo (p < 0.01). This resulted in a pmel CD8+ T cell/Treg ratio of >10 with combination immunotherapy compared to ratios of 3, 1.3, and 0.7 for anti-CTLA-4 only, IL-2 only, and placebo treatments, respectively.

## Conclusions

Combination immunotherapy with anti-CTLA-4 and IL-2 increases the tumor-specific CD8+ T cell/regulatory CD4+ T cell ratio in the tumor microenvironment and significantly decreases tumor growth (compared to monotherapy alone or placebo). These findings propose a previously unrecognized mechanism for anti-CTLA-4 in which regulatory CD4+ T cells are redirected out of the tumor microenvironment into the tumor-draining lymph nodes. These data highlight the potential synergistic action of anti-CTLA-4 combined with IL-2 for the treatment of melanoma.

